# Autoimmune inner ear disease in a melanoma patient treated with pembrolizumab

**DOI:** 10.1186/s40425-016-0114-4

**Published:** 2016-02-16

**Authors:** Matthew Zibelman, Natasha Pollak, Anthony J Olszanski

**Affiliations:** Department of Medical Oncology, Fox Chase Cancer Center, Temple Health, 333 Cottman Avenue, Philadelphia, PA 19111 USA; Department of Otolaryngology-Head & Neck Surgery, Temple University School of Medicine, 3401 North Broad Street, Kresge West, Philadelphia, PA 19140 USA

**Keywords:** Melanoma, PD-1 inhibitor, Pembrolizumab, Autoimmune inner ear disease (AIED), Immune-related adverse events (irAEs), Hearing loss

## Abstract

**Background:**

Immune related adverse events affecting various organ systems are a recognized potential consequence of immune checkpoint inhibition. However, autoimmune inner ear disease is one complication not previously associated with the use of checkpoint inhibitors, though it has been reported after adoptive cell immunotherapy.

**Case Presentation:**

Here we present what we believe is the first case of autoimmune inner ear disease resulting from treatment with an immune checkpoint inhibitor in a patient with metastatic melanoma. An 82 year old male presented with widespread metastatic mucosal melanoma and was initially treated with the CTLA-4 inhibitor ipilimumab but had progression of disease after four doses. He was subsequently treated with the PD-1 inhibitor pembrolizumab and after two doses the patient noted bilateral hearing loss. Otology evaluation was significant for the development of bilateral sensorineural hearing loss and the patient was started on treatment with bilateral intratympanic dexamethasone injections. He experienced significant recovery of his hearing deficit with the intratympanic injections and restaging imaging after 12 weeks of pembrolizumab demonstrated a dramatic reduction in tumor burden.

**Conclusion:**

Autoimmune inner ear disease has been previously associated with the therapeutic transfer of genetically engineered lymphocytes as an on-target effect of donor T-cells recognizing antigens on cells in the inner ear. It is important for physicians to have a high clinical index of suspicion for the appropriate recognition and management of any potential autoimmune toxicity with checkpoint inhibitors given the variability of presentation and unique aspects of toxicity.

**Electronic supplementary material:**

The online version of this article (doi:10.1186/s40425-016-0114-4) contains supplementary material, which is available to authorized users.

## Background

Immunotherapy with the immune checkpoint inhibitors (ICIs) has ushered in a new age of promise and possibility in the treatment of metastatic melanoma. However, unlike more traditional cytotoxic chemotherapy agents, ICIs can incite inflammatory states in any organ system and the resultant toxicities have been termed immune-related adverse events (irAEs). The most common irAEs reported across trials for the various agents have included dermatitis, colitis, hepatitis, and various endocrinopathies, but other less common but serious irAEs have been reported [1–5]. Fortunately, it has been clearly established that prompt recognition of irAEs, followed by the judicious application of anti-inflammatory agents administered in accordance with established treatment algorithms, can significantly reduce the burden of these toxicities [2]. Here we report a case of autoimmune inner ear disease (AIED), occurring in association with the anti-programmed death 1 (PD-1) antibody pembrolizumab in a patient with metastatic melanoma, that improved markedly after intratympanic dexamethasone injections.

## Case presentation

**Fig. 1:**
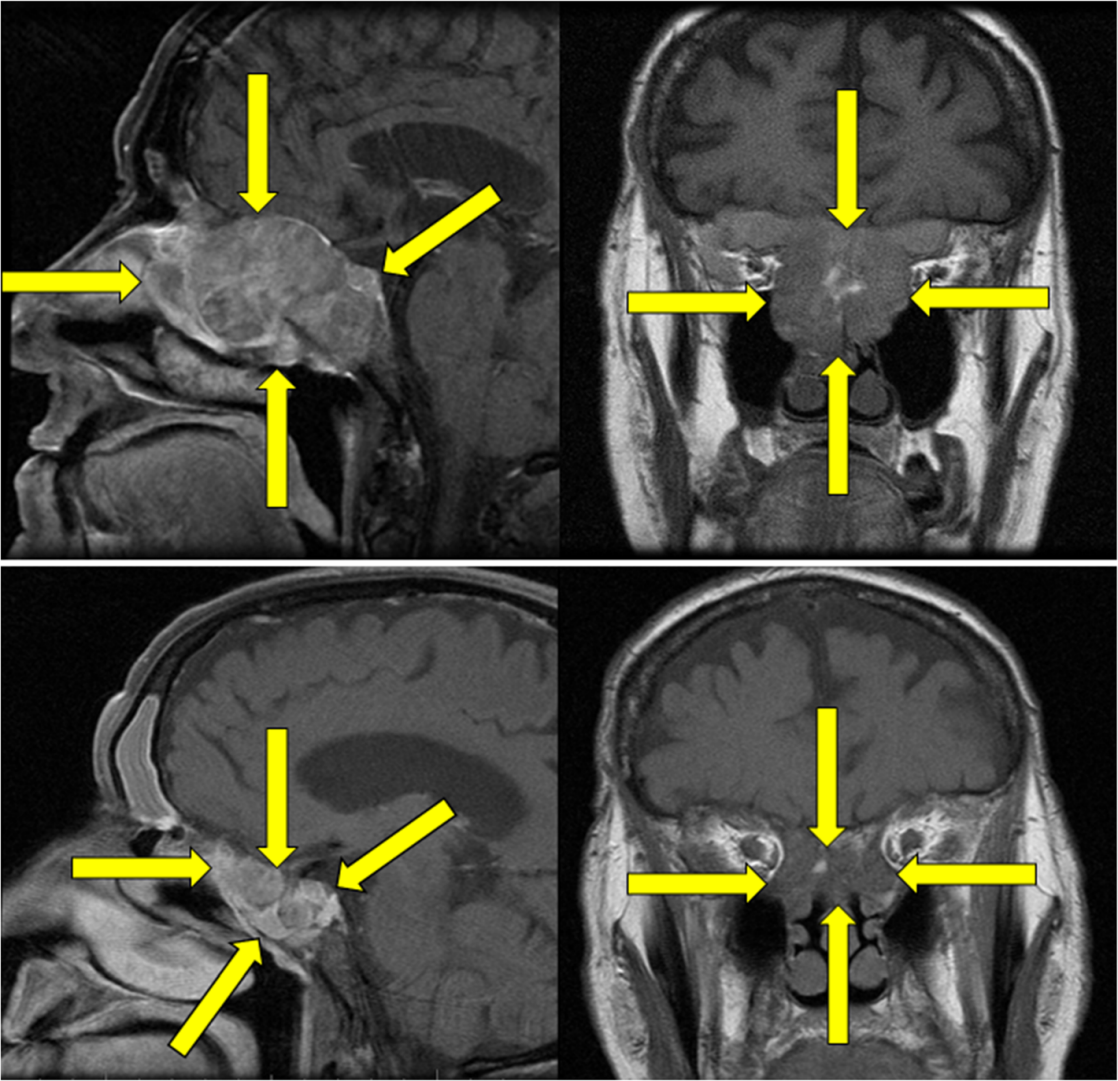
Baseline brain MRI images showing initial primary mucosal melanoma lesion. Primary melanoma centered in the sphenoethmoidal sinuses. Representative sagittal and coronal T1 images are displayed. The upper boxes correspond to the initial MRI, while the lower boxes reflect post-treatment images. Yellow arrows denote approximate tumor boundaries

An 82 year old male was initially evaluated for complaints of nasal fullness, followed by a sensation of “bulging” eyes associated with blurred vision and xerophthalmia. Ocular exam at that time was unremarkable, with impression of mild exophthalmos. Computed tomography (CT) scan of the sinuses revealed a large soft tissue lesion in the posterior nasal cavity and bilateral ethmoid air cells with erosion through the lamina papyracea and extension into the posterior superior aspects of bilateral orbits. Additionally, the optic canals appeared to harbor tumor growth radiologically, and there was soft tissue opacification of the sphenoid bone with erosion into the clivus. Magnetic resonance imaging (MRI) of the orbits delineated an expansile mass within the sphenoethmoidal region and extending posteriorly to invade the clivus, superiorly and posteriorly into the preoptic chiasm with compression of the optic tracts, and associated bilateral proptosis. There was no evidence of parenchymal brain metastases. The upper boxes in Fig. 1 show representative images of the patient’s baseline MRI.

Nasal endoscopy with biopsy revealed a necrotic mass in the posterior right nasal cavity extending down towards the floor of the nose. Pathology from the biopsy specimens was consistent with a diagnosis of primary mucosal melanoma of the ethmoid sinus. Subsequent mutational analysis revealed an NRAS mutation (p.Q61K) but wild type BRAF and cKIT. Complete staging with a positron emission test (PET)/CT scan demonstrated the primary tumor in the nasal cavity, as well as a second active lesion in the right posterior maxillary sinus. Additionally, he was found to have active lesions in the right lower lobe of the lung, left inferior mediastinum, left retrocrural space, multiple liver and mesenteric lesions, bilateral adrenal involvement and diffuse bony involvement.

The patient was referred to medical oncology at which time he noted progressive facial fullness with diplopia causing difficulty ambulating without assistive devices and a 12 lb weight loss. Laboratory studies were significant for a hemoglobin of 12.1 gm/dL, a creatinine of 1.67 mg/dL (baseline 1.5–1.7 mg/dL), a markedly elevated lactate dehydrogenase (LDH) of 3317 u/L (normal range 313–618 u/L) and an alkaline phosphatase of 142 u/L (38–126 u/L). Initial treatment options considered included systemic therapy and localized radiotherapy to the intracranial mass for palliation. Given the lack of survival data with radiation and the large treatment field that would be involved, the decision was made with the patient to proceed with systemic therapy. Treatment was initiated with the cytotoxic T-lymphocyte associated protein 4 (CTLA-4) inhibitor ipilimumab at 3 mg/kg and the patient received four doses over 12 weeks. Fatigue and anorexia were the only notable side effects. His LDH remained high, increasing to 4128 u/L on the day of the third dose of ipilimumab, though it decreased to 2606 u/L on the date of the fourth dose. His alkaline phosphatase peaked by the 4^th^ dose at 351 u/L. A restaging CT scan of the chest, abdomen, and pelvis after four doses of ipilimumab showed progression of disease with development of new bilateral lung nodules, growth in the liver lesions, and evidence of innumerable mesenteric and peritoneal nodules consistent with peritoneal carcinomatosis. He was immediately started on second line therapy with the PD-1 inhibitor pembrolizumab at 2 mg/kg given every 3 weeks.

At follow-up evaluation 3 weeks after his first dose of pembrolizumab the patient noted an improved energy level and increased appetite but still was experiencing diplopia. His LDH and alkaline phosphatase had decreased dramatically to 658 u/L and 172 u/L, respectively, and he received a second dose of pembrolizumab. Two weeks later, the patient reported sudden onset of bilateral hearing loss and was referred to otology for an evaluation. An audiogram revealed mild to moderately severe symmetric sensorineural hearing loss with word recognition scores of 48 % in the right ear and 44 % in the left ear. Without a recent offending agent such as ototoxic chemotherapy or infectious meningitis, consideration was given that his sudden onset bilateral hearing loss could be autoimmune mediated. A trial of intratympanic dexamethasone injections was initiated with twice weekly injections, initially in the right ear and then in both ears. Dexamethasone solution (10 mg/mL) was injected through a myringotomy and the middle ear cleft filled with the solution. The patient then remained recumbent with the treated ear up for 20 min to allow diffusion of the dexamethasone through the round window membrane into the basal turn of the cochlea. After four intratympanic dexamethasone injections on the right and two on the left, the patient noted marked improvement in hearing. He continued to receive pembrolizumab 2 mg/kg every 3 weeks and never required systemic steroids.

**Fig. 2:**
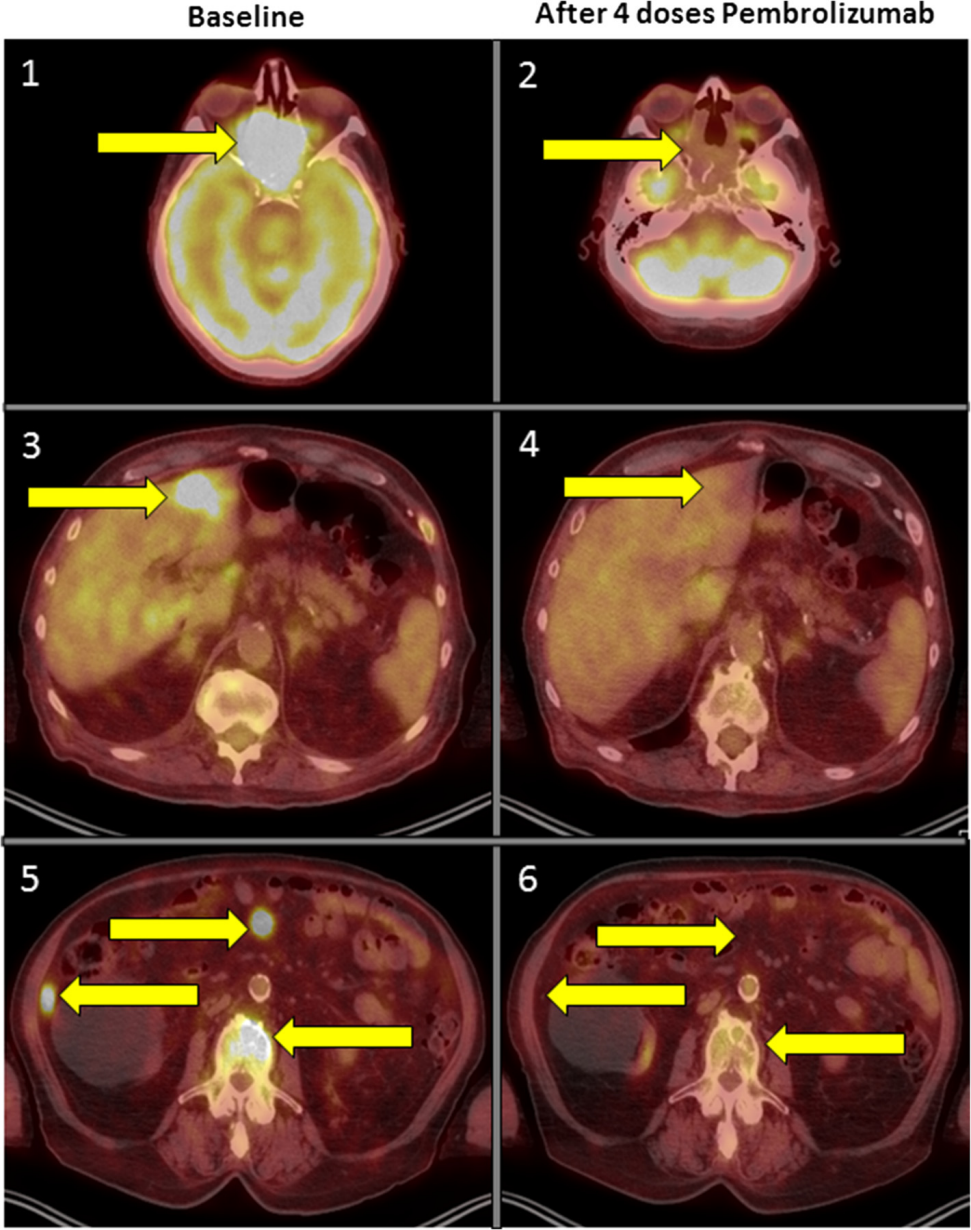
Baseline (left column) and post-pembrolizumab (right column) therapy representative PET/CT images. Side-by-side comparative images of PET/CT scan at baseline and after the first four doses of pembrolizumab. Yellow arrows denote sites of active or previously identified melanoma deposits. (1 and 2) Primary sphenoethmoidal mass showing a CR after treatment. (3 and 4) CR in a left hepatic lobe liver metastasis. (5 and 6) CR in two intra-abdominal metastatic implants and in a vertebral metastasis

Repeat PET/CT was performed after four doses of pembrolizumab and representative images are shown in Fig. 2 compared to the baseline PET/CT scan. The restaging images demonstrated significant improvement at all known sites, with markedly reduced activity of the primary mass and near complete response in all lesions in the chest, abdomen, and bones. His LDH remained in the normal range. After receiving a total of six intratympanic dexamethasone injections in the right ear and four in the left, the patient’s hearing returned subjectively to his baseline. Post-injection audiogram showed normal to moderately severe symmetric sensorineural hearing loss with word recognition scores 88 % in the right ear and 84 % in the left ear and his hearing had significantly improved at low to mid frequencies but not at high frequencies (original audiogram image available, see Additional file 1). He had no other side effects from therapy. At the time of manuscript preparation, the patient continued on treatment with pembrolizumab 2 mg/kg every three weeks and was being monitored with serial audiograms for his sensorineural hearing loss. He reported minimal improvement in the impaired visual acuity that occurred secondary to direct tumor invasion of the optic tracts at the time of his referral. A follow-up MRI of his brain to reassess the primary tumor mass in the paranasal sinuses correlated with the response noted in the PET/CT and representative images compared to the original MRI are available in Fig. 1.

## Discussion

While irAEs can affect any organ of the body, this case represents the first report of causally-related AIED attributed to an ICI. However, treatment-related autoimmunity affecting the inner ear and leading to audiovestibular symptoms has been previously described with adoptive cell immunotherapy (ACI) after therapeutic transfer of genetically engineered lymphocytes. A 2009 study using tumor-specific autologous T-cells genetically modified to have a high specificity for melanoma/melanocyte antigens MART-1 or gp100 were generated and administered to 36 patients with metastatic melanoma [6]. Fifteen patients developed some detectable hearing loss (10/20 and 5/16 in the MART-1 and gp100 cohorts, respectively). Of this group, seven patients received intratympanic corticosteroid injections and all had at least some improvement. Nine of the 36 patients also manifested some modicum of vestibular dysfunction that responded to treatment. A second prospective study was performed with a similar cohort of refractory metastatic melanoma patients who also underwent ACI with genetically modified T-cells [7]. In this study, 32 patients underwent ACI and received serial audiometric testing along with symptom-initiated vestibular function testing. Audiometric testing established that 17 patients (53.1 %, 9 mild/8 moderate) experienced some objective hearing loss, while seven patients (22 %) had confirmed vestibular dysfunction. All but one of the patients with mild hearing loss recovered to pre-treatment function, while amongst those with moderate hearing loss, half returned to their pre-treatment level, and the other half experienced a partial recovery. Three of the seven patients with vestibular symptoms regained normal vestibular function. Overall, ten patients received intratympanic steroid injections (mild deficit: 2; moderate deficit: 7; vestibular dysfunction: 1), with complete recovery in six patients and four patients with moderate hearing loss achieving partial recovery. The study noted that the number of infused T-cells correlated with the onset and degree of audiovestibular defect.

AIED after ACI as described is postulated to be an on-target effect of the genetically-modified T-cells generating an autoimmune response after recognition of antigens found on melanocytic cells in the striae vascularis of the inner ear [6–8]. The majority of patients in these studies also developed other autoimmune effects, most notably rash and uveitis. This constellation of symptoms is similar to the Vogt-Koyanagi-Harada syndrome, a granulomatous autoimmune disease characterized by bilateral posterior uveitis along with poliosis, vitiligo, central nervous system deficits, and hearing loss that is thought to be caused by a T-cell mediated autoimmune destruction of melanocytes [9, 10]. Our patient was treated sequentially with ipilimumab followed by pembrolizumab after disease progression. The onset of AIED during ICI therapy for melanoma, along with the rapid and impressive improvement after intratympanic steroid injections, is highly suggestive of an irAE. Although a pretreatment baseline audiogram that predates the melanoma treatment was not performed, the permanent sensorineural hearing loss at high frequencies is most likely attributable to long-standing cochlear hair cell damage due to presbycusis or prior noise exposure, rather than AIED. This type of hearing loss is permanent and is not expected to improve with intratympanic dexamethasone injections. Indeed, both the patient and his wife felt that his hearing had returned to his baseline.

Given the onset of the hearing deficit during therapy with pembrolizumab, and the remarkable response the patient had to this drug, we speculate that the likely mechanism of hearing loss is secondary to a cross-reactive autoimmune response of the patient’s T-cells to melanocytes in the inner ear. Activated T-cells recognizing particular melanocytic antigens may have been liberated after pembrolizumab–induced PD-1 inhibition and directed a tumor antigen specific anti-melanoma response that was effective in eradicating his metastatic disease, but recognition of analogous antigens on cells in the inner ear resulted in the hearing loss. However, it is important to note that delayed irAEs and responses have been reported with ICI therapy, so whether the prior use of ipilimumab in this patient could have been a contributing factor in the development of his hearing loss (or subsequent efficacy) remains unknown.

## Conclusions

In summary, we report a case of an 82 year old male with metastatic melanoma who developed hearing loss while receiving ICI therapy with pembrolizumab that dramatically improved with intratympanic steroid injections. We believe that this represents the first reported case of temporally-related AIED dysfunction resulting from ICI therapy. Notwithstanding this case, we do not routinely assess baseline audiograms for patients receiving ICI therapy, and given the apparent rarity of this irAE, we do not recommend such. Nonetheless, given the increasing use of ICIs across a spectrum of oncologic diseases, and the recent approval of ipilimumab in combination with nivolumab in patients with melanoma, a study to determine the frequency of autoimmune hearing dysfunction is warranted and is being planned. As ICI use becomes increasingly widespread, this case highlights the importance of maintaining vigilance in monitoring for potential irAEs and promptly employing systemic, or in some cases local, corticosteroids for treatment-related adverse events.

## Informed consent

Written informed consent was obtained from the patient for publication of this case report and any accompanying images. A copy of the written consent is available for review by the Editor-in-Chief of this journal.

## Additional file

Additional file 1:
**Original audiogram report showing pure tone and speech audiometry.** Caption/Description of data: After a series of intratympanic dexamethasone injections, hearing thresholds improved significantly in both ears in the 250 – 2000 Hz range. Left: Pre and post injection audiometry tracings. Black line denotes pre-injection hearing thresholds and red line denotes post-injection thresholds. Right: Word recognition scores. Right ear word recognition improved from 48 to 88 %, and left ear WR improved from 44 to 84 %. (JPG 1002 kb)

## Abbreviations

ACI: adoptive cell immunotherapy
AIED: autoimmune inner ear disease
CT: computed tomography
FDA: Food and Drug Administration
ICI: immune checkpoint inhibitor
irAE: immune-related adverse events
LDH: lactate dehydrogenase
MRI: magnetic resonance imaging
OS: overall survival
PD-1: Programmed Death 1
PET: positron emission test
