# Supplementary figures and images for: Autoimmune inner ear disease in a melanoma patient treated with pembrolizumab
Source: J Immunother Cancer. 2016 Feb 16;4:8. doi: 10.1186/s40425-016-0114-4 (PMC4754989; doi:10.1186/s40425-016-0114-4)

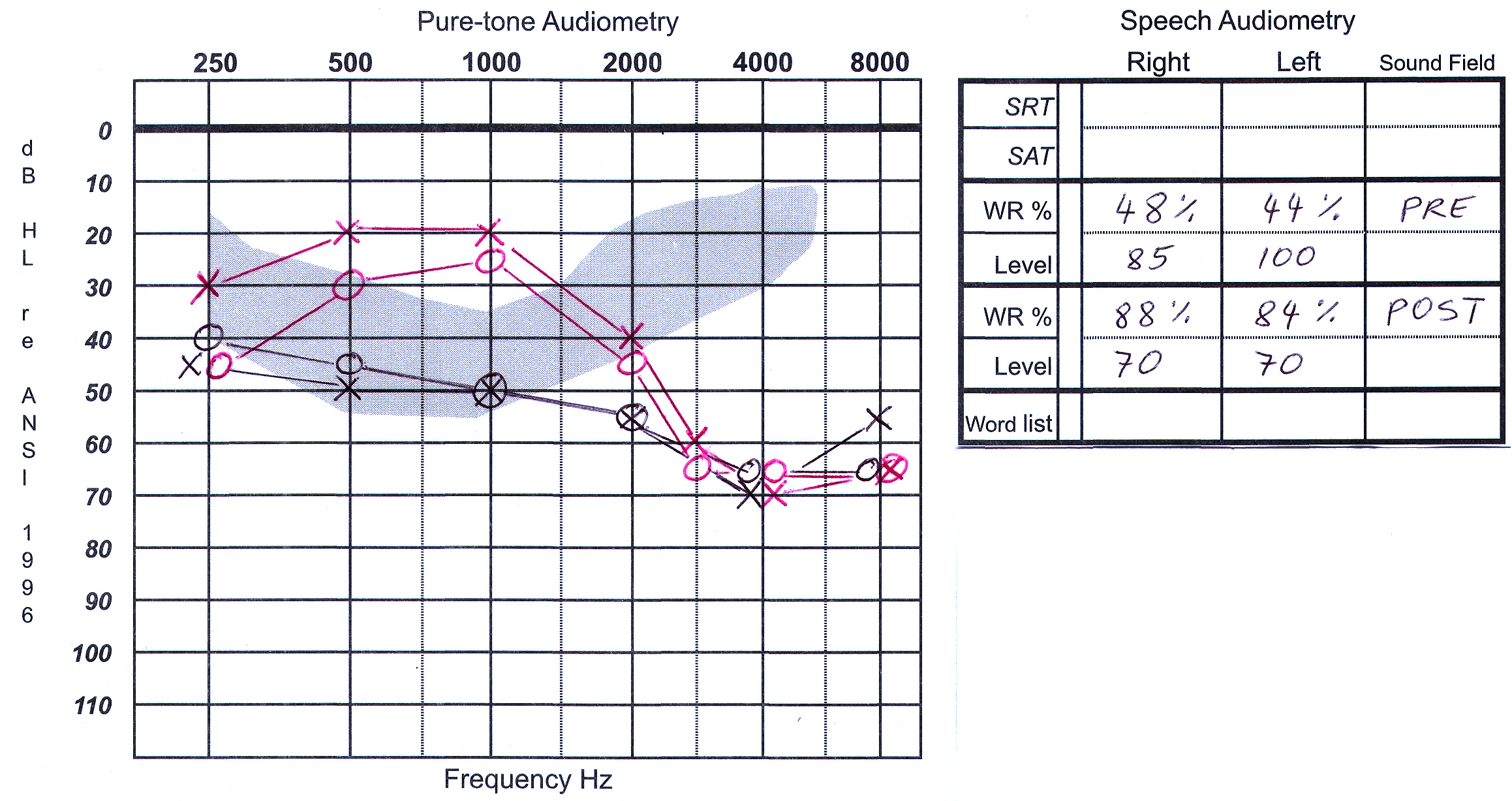

Supplement: Additional file 1: — Original audiogram report showing pure tone and speech audiometry. Caption/Description of data: After a series of intratympanic dexamethasone injections, hearing thresholds improved significantly in both ears in the 250 – 2000 Hz range. Left: Pre and post injection audiometry tracings. Black line denotes pre-injection hearing thresholds and red line denotes post-injection thresholds. Right: Word recognition scores. Right ear word recognition improved from 48 to 88 %, and left ear WR improved from 44 to 84 %. (JPG 1002 kb) [file 40425_2016_114_MOESM1_ESM.jpg]
